# Identification of Ten Core Hub Genes as Potential Biomarkers and Treatment Target for Hepatoblastoma

**DOI:** 10.3389/fonc.2021.591507

**Published:** 2021-04-01

**Authors:** Rui Sun, Simin Li, Ke Zhao, Mei Diao, Long Li

**Affiliations:** ^1^ Department of Pediatric Surgery, Capital Institute of Pediatrics, Beijing, China; ^2^ Chinese Academy of Medical Sciences and Peking Union Medical College, Beijing, China; ^3^ Stomatological Hospital, Southern Medical University, Guangzhou, China; ^4^ Department of Ophthalmology, Ningbo Hangzhou Bay Hospital, Ningbo, China

**Keywords:** hepatoblastoma, gene expression omnibus, random forest classifier, nomogram, diagnosis

## Abstract

**Background:**

This study aimed to systematically investigate gene signatures for hepatoblastoma (HB) and identify potential biomarkers for its diagnosis and treatment.

**Materials and Methods:**

GSE131329 and GSE81928 were obtained from the Gene Expression Omnibus (GEO) database. Differentially expressed genes (DEGs) between hepatoblastoma and normal samples were identified using the Limma package in R. Then, the similarity of network traits between two sets of genes was analyzed by weighted gene correlation network analysis (WGCNA). Cytoscape was used to visualize and select hub genes. PPI network of hub genes was construed by Cytoscape. GO enrichment and KEGG pathway analyses of hub genes were carried out using ClueGO. The random forest classifier was constructed based on the hub genes using the GSE131329 dataset as the training set, and its reliability was validated using the GSE81928 dataset. The resulting core hub genes were combined with the InnateDB database to identify the innate core genes.

**Results:**

A total of 4244 DEGs in HB were identified. WGCNA identified four modules that were significantly correlated with the disease status. A total of 114 hub genes were obtained within the top 20 genes of each node rank. 6982 relation pairs and 3700 nodes were contained in the PPI network of 114 hub genes. GO enrichment and KEGG pathway analyses of hub genes were focused on MAPK, cell cycle, p53, and other crucial pathways involved in HB. A random forest classifier was constructed using the 114 hub genes as feature genes, resulting in a 95.5% true positive rate when classifying HB and normal samples. A total of 35 core hub genes were obtained through the mean decrease in accuracy and mean decrease Gini of the random forest model. The classification efficiency of the random forest model was 81.4%. Finally, *CDK1*, *TOP2A*, *ADRA1A*, *FANCI*, *XRCC1*, *TPX2*, *CCNB2*, *CDK4*, *GLYATL1*, and *CFHR3* were identified by cross-comparison with the InnateDB database.

**Conclusion:**

Our study established a random forest classifier that identified 10 core genes in HB. These findings may be beneficial for the diagnosis, prediction, and targeted therapy of HB.

## Introduction

Hepatoblastoma (HB) is the most common pediatric liver tumor, affecting mainly children under 4 years of age (1). Although its incidence has increased markedly over the last few decades, HB is a rare pediatric malignancy with an annual incidence of 1.5 cases per million (2). Complete surgical resection and chemotherapy have contributed to improving the survival rate of up to 80% in all diagnosed patients (3). However, the prognosis for patients with clinically advanced HB remains relatively low. Furthermore, surviving patients can suffer severe and lifelong side effects due to chemotherapy and immunosuppression (4). Lacking of an effective means of early diagnosis is the main reason contributed to the relative worse prognosis for patients with HB. At present, clinicians rely primarily on clinical symptoms, imaging, and alpha-fetoprotein levels to diagnose the disease. Among these methods, no novel biomarker had been showed except the conventional AFP levels. However, the sensitivity and specificity were not satisfied due to the various sources of AFP from different patients.In the previous study of Liu et al, it claimed there were 5 patients with a normal AFP level were diagnosed as HB (5). Consequently, novel biomarkers must be identified to develop efficient diagnostic methods and therapeutic strategies for patients affected with HB.

Recent studies have demonstrated that some RNAs are aberrantly expressed in HB thanks to the advancements in gene chips and high-throughput sequencing. A recent study reported by Liu et al. revealed that the increase of N6-Methyladenosine modification is an oncogenic mechanism in HB (6). Multiple studies have also shown that different genes, including genes encoding for long non-coding RNAs, are involved in the proliferation, apoptosis, and glutaminolysis of HB, such as zinc finger antisense 1 (7), 3-hydroxy-3-methylglutaryl-CoA synthase 1 (8), and TUG1 (9). Since the analysis pipeline, experimental methods, and sample size of each research are different, the conclusions have been controversial. Thus, a further bioinformatics exploration of data published in public databases could consolidate data and reveal novel additional genes associated with HB.

In this study, we investigated two HB datasets obtained from the Gene Expression Omnibus (GEO) database to identify reliable differentially expressed genes (DEGs) in HB. Through deep and comprehensive bioinformatics analysis, we identified hub genes, which we used to construct a diagnosis classification for HB. Moreover, we identified the core genes using our classification and cross-comparing it with the congenital immune-related genes present in the InnateDB database. The identification of a list of core genes may provide new diagnostic, prognostic, and potential therapeutic biomarkers for HB.

## Materials and Methods

### Acquisition of Microarray Profiles

**Figure 1:**
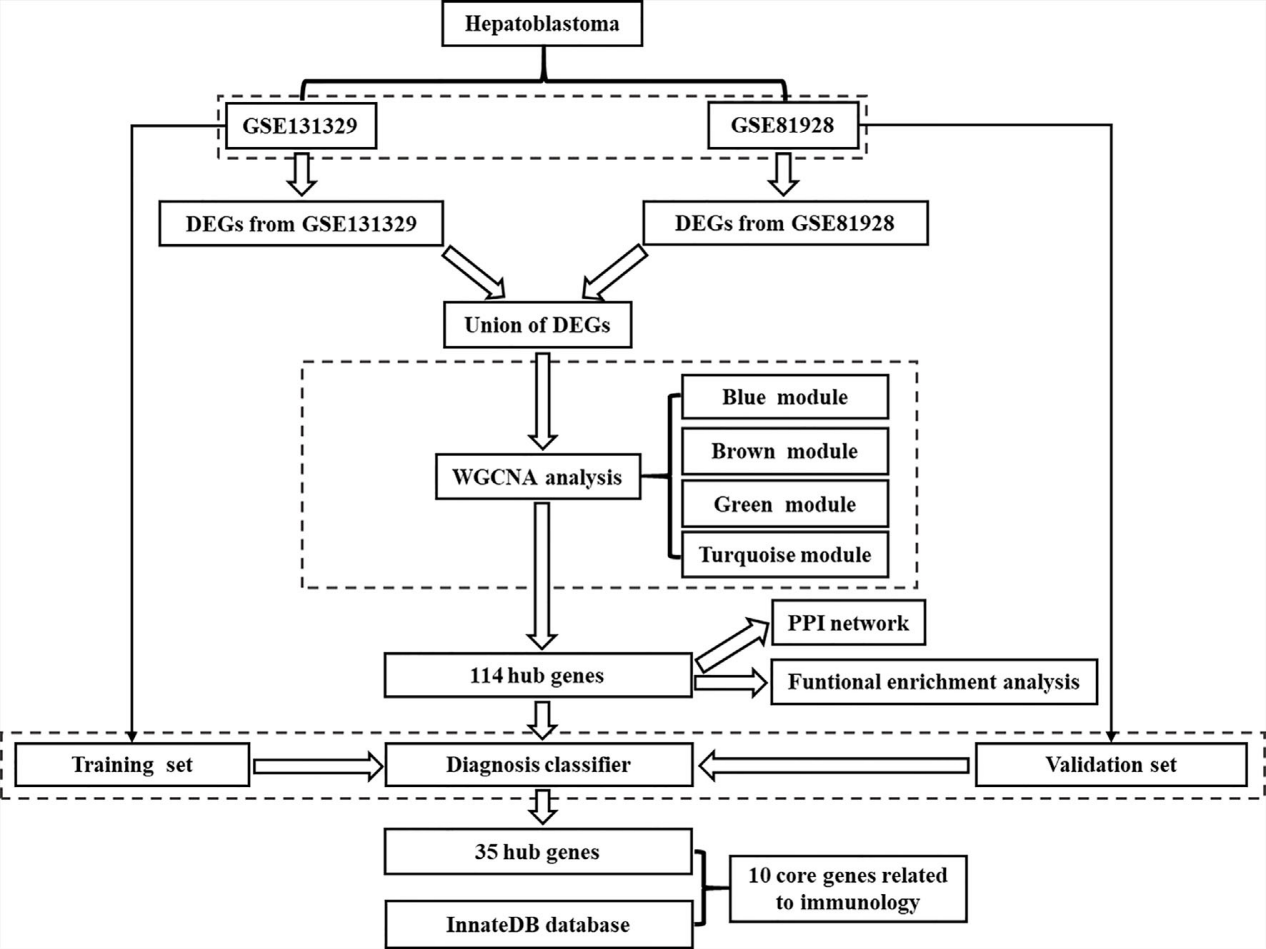
Flowchart showing the protocol of the study. DEGs, differentially expressed genes; WGCNA, Weighted gene correlation network analysis; PPI, protein-protein interaction.

The flow chart for the study was showed in **Figure 1**. Microarrays that met the following criteria were collected: (1) studies including at least 20 samples and (2) examination expression of both cancerous tissue and adjacent noncancerous tissue from HB patients. Microarrays without useful data for analysis were excluded. Finally, 2 independent microarrays data, GSE131329 and GSE81928 databases, were obtained from the GEO database (http://www.ncbi.nlm.nih.gov/geo). The characteristics of the 2 datasets were presented in **Table 1**. Probes were converted into the corresponding gene symbols according to the annotation information in the dataset.

**Table 1:** The characteristics of the 2 datasets in the study.

Datesets	Country	Researchers/ References	Experiment type	Tumor site	Sample size (normal/tumor)	Platform
GSE131329	Japan	Contributed by Hiyama E, et al.	Expression profiling by array	hepatoblastoma	67 (14/53)	GPL6244 [HuGene-1_0-st] Affymetrix Human Gene 1.0 ST Array
GSE81928	USA	(10)	Expression profiling by high throughput sequencing	hepatoblastoma	26 (3/23)	GPL16791 Illumina HiSeq 2500

Since GSE131329 is chip data and GSE81928 is sequencing data, we used different procedures to deal with 2 datasets. For the GSE131329 dataset, platform annotation files were used to match probes to the gene symbol. If multiple probes matched a single gene, the median ranking value was used as the expression value. Then, the disease and normal gene expression spectrum of GSE131329 was constructed. For the GSE81928 dataset, we excluded from the analysis genes whose expression value was 0 in 80% of the samples. We analyzed a total of 17920 genes from the two data sets, which were then used for the subsequent analyses.

### Identification of Differentially Expressed Genes (DEGs)

The Limma package in R was used to identify DEGs between HB and non-tumor samples. The cutoff value was set to |Log_2_FC (fold-change)| > 0.58 in both datasets to obtain more DEGS for further analysis in accordance with protocols of previous studies (11, 12). Because the experimental assay and platform of the 2 datasets were different, the P value was < 0.05 for GSE131329, and was < 0.01 for GSE81928 to obtain more significant DEGs, which was used the previous researches as reference (13, 14).

### Weighted Gene Correlation Network Analysis (WGCNA)

WGCNA is a systematic biological method used to describe gene association patterns among different samples (15). It can be used to identify highly collaborative gene sets and to identify candidate biomarker genes or therapeutic targets based on gene set interconnection and the correlation between gene sets and phenotypes. Using the GSE131329 dataset as reference, the potential DEGs expression profile of HB was constructed. Then, we identified the related modules of HB, and analyzed the relationship between those modules and either HB or normal samples, using the WGCNA package in R. The identified network of HB modules was visualized using Cytoscape v. 3.8.0 (https://cytoscape.org/) to identify the hub genes in each module.

### Protein-Protein Interaction (PPI) Network Construction of Hub Genes

In order to analyze the role of 114 hub genes in the global human biological network, we constructed a PPI network of modular genes. We downloaded and integrated human interaction protein data from the following database: HPRD release9 (http://www.hprd.org/), IntAct (http://www.ebi.ac.uk/intact/), MINT (http://mint.bio.uniroma2.it/mint/Welcome.do), BioGRID Release 3.4.132 (http://thebiogrid.org/), DIP (http://dip.doe-mbi.ucla.edu/dip/Main.cgi), String (https://string-db.org). We extracted 114 protein interaction pairs of hub genes from the integrated human interaction protein pairs. Even if there was only one protein interacting with one of the 114 module genes, it would be extracted. The PPI network of these 114 modules was visualized by Cytoscape. In the network, 114 hub genes were marked with the color of their modules. Network analyzer, a Cytoscape tool, was used to calculate network topology properties.

### Bioinformatic Analysis of Hub Genes

Gene ontology (GO) analysis was used to identify potential biological processes, cellular components, and molecular functions associated with DEGs. The Kyoto Encyclopedia of Genes and Genomes (KEGG) is a collection of databases for the systematic analysis of gene functions that link genomic information with higher-order functional information (16). GO enrichment and KEGG pathway analysis of the top 20 DEGs in HB were revealed using the ClueGO software. ClueGO software is a Cytoscape App that extracts representative functional biological information from a large list of genes or proteins (17). P < 0.05 was regarded as the cut-off criterion with statistic difference.

### Construction and Validation of the HB Classifier

Random forest is a classification method that uses multiple trees to train and predict samples and is characterized by high accuracy (18). Therefore, we constructed a random forest model for HB, using GSE131329 as the training set, the top 20 genes in the module as the classification feature, and disease and normal samples as the variables. Then, we validated the model using the GSE81928 dataset as an independent validator. The model feature files of training set (**Supplementary Table 1**) and verification set (**Supplementary Table 2**) were shown in the **Supplementary**.

### Cross-Comparison of Biological Markers of HB in InnateDB

InnateDB (http://www.innatedb.com) is a publicly available database of genes, proteins, and experimentally verified interacttions and signaling pathways involved in innate immunity (19). We intersected hub genes related to immunity in HB as revealed by our bioinformatics analysis with genes present in the InnateDB database.

## Results

### Identification of DEGs

**Figure 2:**
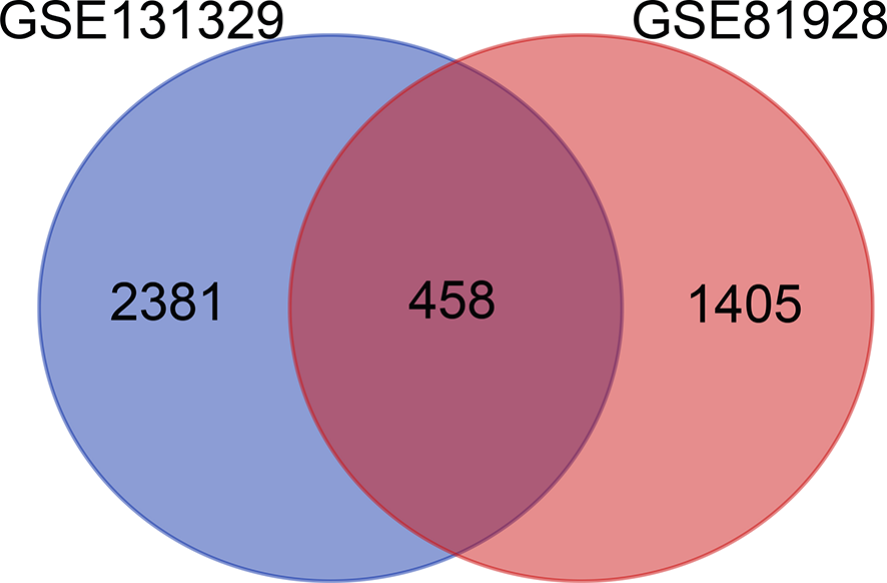
Total of 4,244 DEGs identified from 2 datasets (2839 in GSE131329 and 1863 in GSE81928). DEGs, differentially expressed genes.

**Figure 3:**
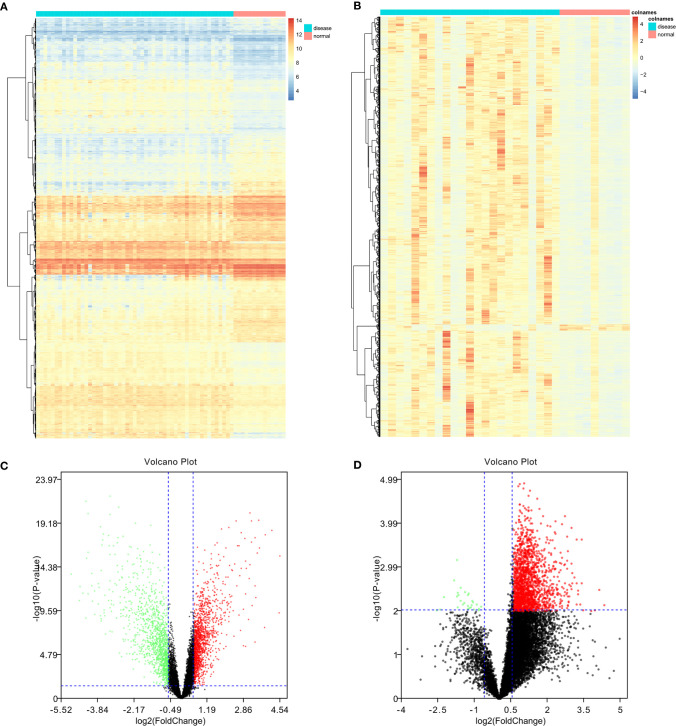
Heat map of differentially expressed genes (DEGs) and volcano plot of genes in the two datasets. Upregulated DEGs are shown in red; downregulated DEGs are shown in green; non-DEGs are shown in black. **(A, B)** GSE131329 dataset; **(C, D)** GSE81928 dataset.

In total, 4244 DEGs (2839 in GSE131329 and 1863 in GSE81928) were identified between tumor and normal tissues (**Supplementary Table 3**), of which in GSE131329, 1368 were downregulated and 1471 were upregulated (**Supplementary Table 4**), while in GSE81928, 28 were downregulated and 1835 were upregulated (**Supplementary Table 5**). There were 453 overlapping DEGs of 2 datasets. The Venn diagrams (Available online: http://bioinformatics.psb.ugent.be/webtools/Venn/) was showed in **Figure 2**. The heat map and Volcano plot of the two datasets were showed in **Figures 3–D**.

### WGCNA

**Figure 4:**
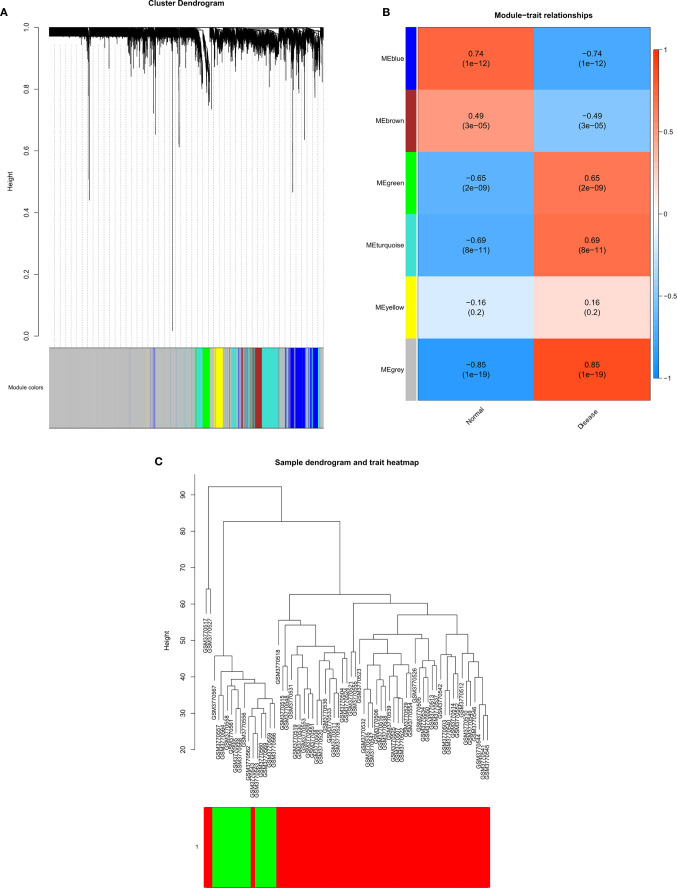
Gene modules identified by WGCNA. **(A)** Cluster dendrogram of the coexpression network modules; **(B)** Gene relation between hepatoblastoma and normal samples; **(C)** Cluster tree of hepatoblastoma and normal samples.

Using the GSE131329 dataset and the WGCNA package in R to analyze the co-expression with default parameters, we constructed the expression spectrum of the 4244 DEGs. We obtained six different modules (indicated in blue, brown, green, turquoise and yellow) (**Figure 4**). The blue, brown, green, and turquoise modules were significantly correlated with HB and normal samples (**Figure 4**). The blue and brown modules were negatively correlated with HB disease, whereas the green and turquoise modules were positively correlated with HB disease. The modules contained 408 genes (blue), 188 genes (brown), 123 genes (green), and 666 genes (turquoise). Sample clustering is shown in **Figure 4**. The red component represents HB samples, while green represents the non-tumor samples.

### Modules Network Construction and Hub Genes Identification

**Figure 5:**
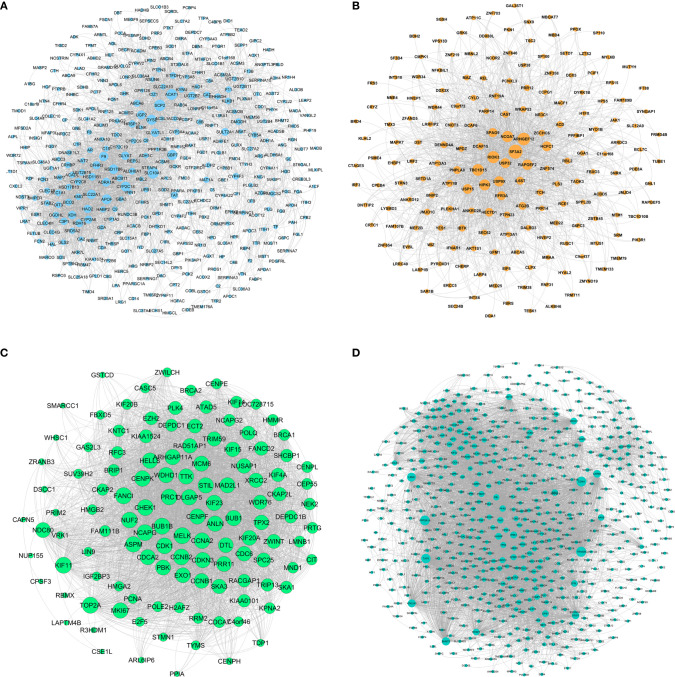
Gene symbols and gene interaction in the four modules, as determined by ClueGO. **(A)** Blue module; **(B)** Brown module; **(C)** Green module; **(D)** Turquoise module.

The network of HB-related modules (blue, brown, green, and turquoise modules) is shown in **Figures 5A–D**. Then, we analyzed the network using Cytoscape, selecting the top 20 genes of each module as the HB hub genes (genes with the same degree were taken out at the same time). Degree refers to the number of connections between one point and other points in the network. We identified a total of 114 hub genes (**Supplementary Table 6**). The larger the point is, the greater the degree of the representative node.

### PPI Network Construction of Hub Genes

**Figure 6:**
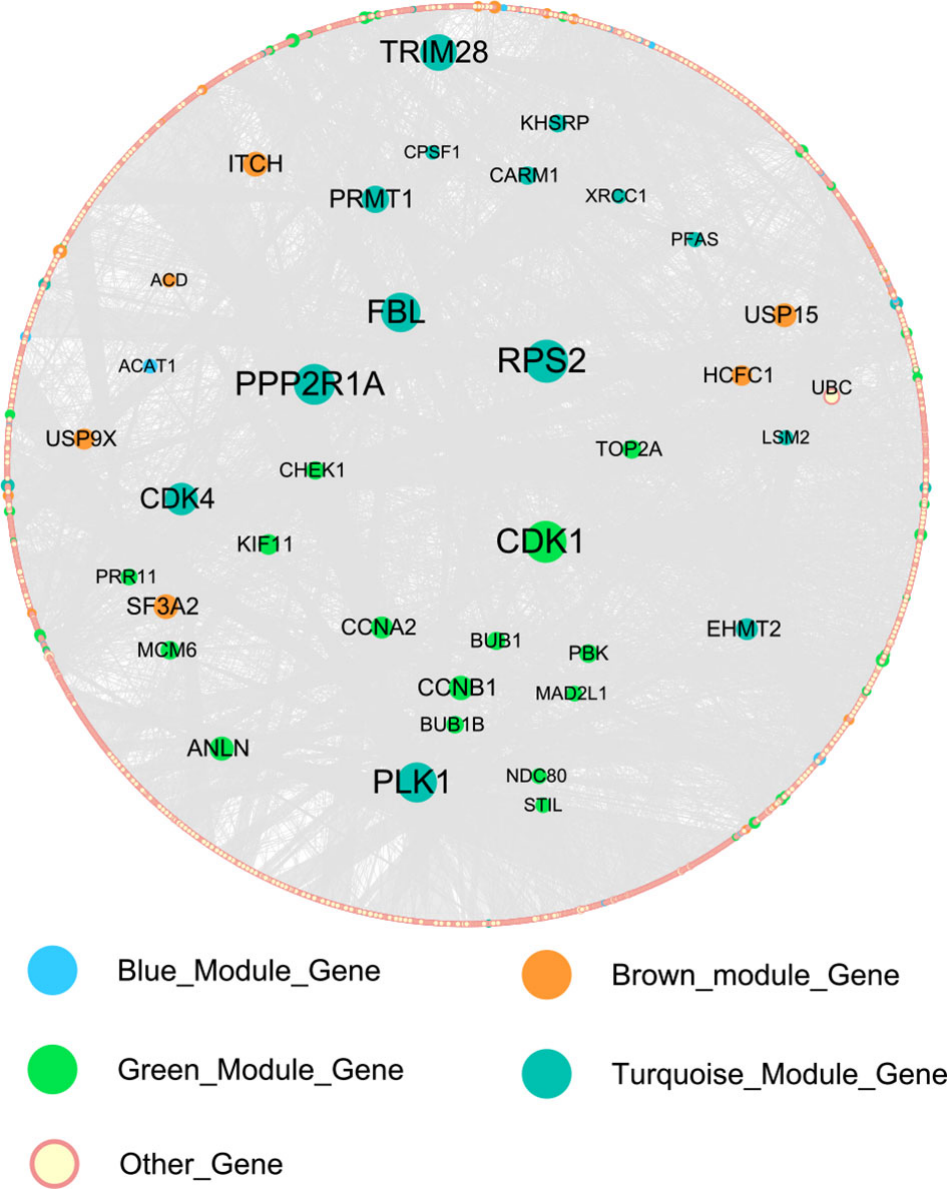
The protein-protein interaction (PPI) network of the 114 hub genes.

We constructed the PPI network of 114 hub genes. Finally, the network was consisted of 6982 relation pairs and 3700 nodes (**Figure 6**). For the topological properties of nodes, we arranged them in descending order according to the interaction degrees, and selected the top 20 genes to display, including RPS2, PPP2R1A, CDK1, FBL, PLK1, TRIM28, CDK4, PRMT1, SF3A2, ITCH, ANLN, USP15, CCNB1, EHMT2, CCNA2, USP9X, HCFC1, KIF11, TOP2A, as shown in **Table 2**. These genes play an important role in the global biological network.

**Table 2:** Network topological characteristic of top 20 nodes in PPI network.

Gene	label	Degree	Average ShortestPath Length	BetweennessCentrality	ClosenessCentrality	ClusteringCoefficient	Stress	TopologicalCoefficient
RPS2	Turquoise	304	2.796578	0.088088	0.35758	0.002084	35651276	0.011482
PPP2R1A	Turquoise	285	2.733026	0.10859	0.365895	0.001112	32731778	0.008927
CDK1	Green	277	2.606464	0.086532	0.383662	0.007953	23033244	0.008038
FBL	Turquoise	272	2.764259	0.083119	0.361761	0.003337	26783624	0.009266
PLK1	Turquoise	271	2.749593	0.087714	0.36369	0.002952	24705604	0.009257
TRIM28	Turquoise	251	2.847094	0.07047	0.351235	0.000829	35607200	0.02181
CDK4	Turquoise	215	2.763987	0.064648	0.361796	0.002478	17600494	0.009869
PRMT1	Turquoise	174	2.833786	0.051374	0.352885	0.003654	15009342	0.013589
SF3A2	Brown	156	2.933188	0.048426	0.340926	0	11812770	0.028122
ITCH	Brown	155	2.893808	0.049782	0.345565	0.000922	10898838	0.016011
ANLN	Green	149	2.937534	0.055011	0.340422	0.000998	7721342	0.020761
USP15	Brown	146	2.891092	0.04818	0.34589	0.001606	9324218	0.015811
CCNB1	Green	144	2.747691	0.02827	0.363942	0.019814	5878754	0.013833
EHMT2	Turquoise	132	2.939164	0.038808	0.340233	0	8115194	0.02823
CCNA2	Green	126	2.771863	0.026821	0.360768	0.01981	4666808	0.015196
USP9X	Brown	125	2.898968	0.034266	0.34495	0.001419	9220858	0.020271
HCFC1	Brown	120	2.885117	0.032388	0.346606	0.003641	7730212	0.018792
KIF11	Green	118	2.847366	0.039087	0.351202	0.001883	5902744	0.013225
TOP2A	Green	106	2.892178	0.020279	0.34576	0.004672	7719634	0.024587

### Bioinformatic Analysis of Hub Genes

**Figure 7:**
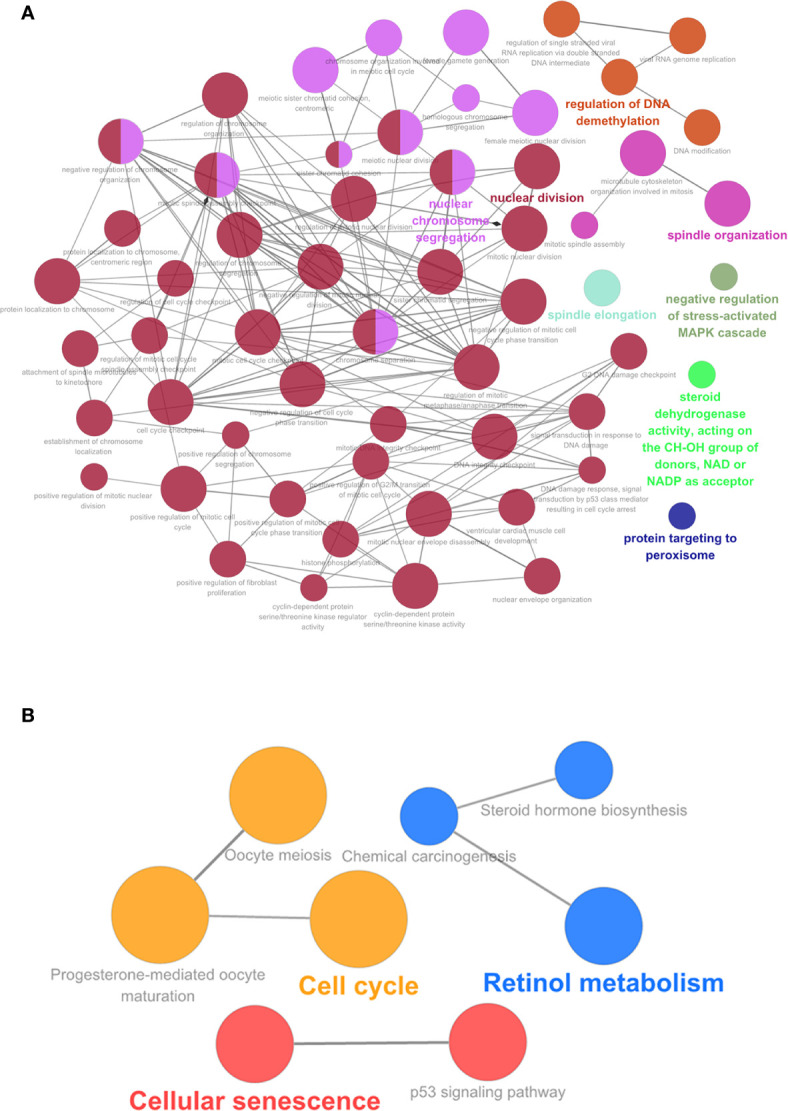
**(A)** GO analysis of the 114 hub genes. **(B)** KEGG pathway of the 114 hub genes, GO.

We performed the GO enrichment and KEGG pathway analysis of the top 20 genes using ClueGO. Of the 114 hub genes identified, 21 were from the blue module, 24 from the brown module, 46 from the green module, and 23 from the turquoise module. GO function enrichment results are shown in **Figure 7**. Hub genes were enriched in multiple biological functions, including regulation of DNA demethylation, nuclear chromosome isolation, protein targeting to the peroxisomes, negative regulation of stress-activated MAPK cascade, signal transduction by p53 class mediator resulting in cell cycle arrest, toroid dehydrogenase activity with the CH-OH group acting as donors and NAD or NADP as acceptors et al. (**Supplementary Table 7**). The KEGG pathway analysis results showed that these hub genes also participated in the P53 signaling pathway, cell aging, cell cycle, meiotic maturation process of oocytes, progesterone-mediated oocyte maturation, steroid biosynthesis, retinol metabolism, chemical carcinogenesis, and other biological pathways (**Figure 7**) (**Supplementary Table 8**).

### Construction and Validation of the HB Classification Method

**Figure 8:**
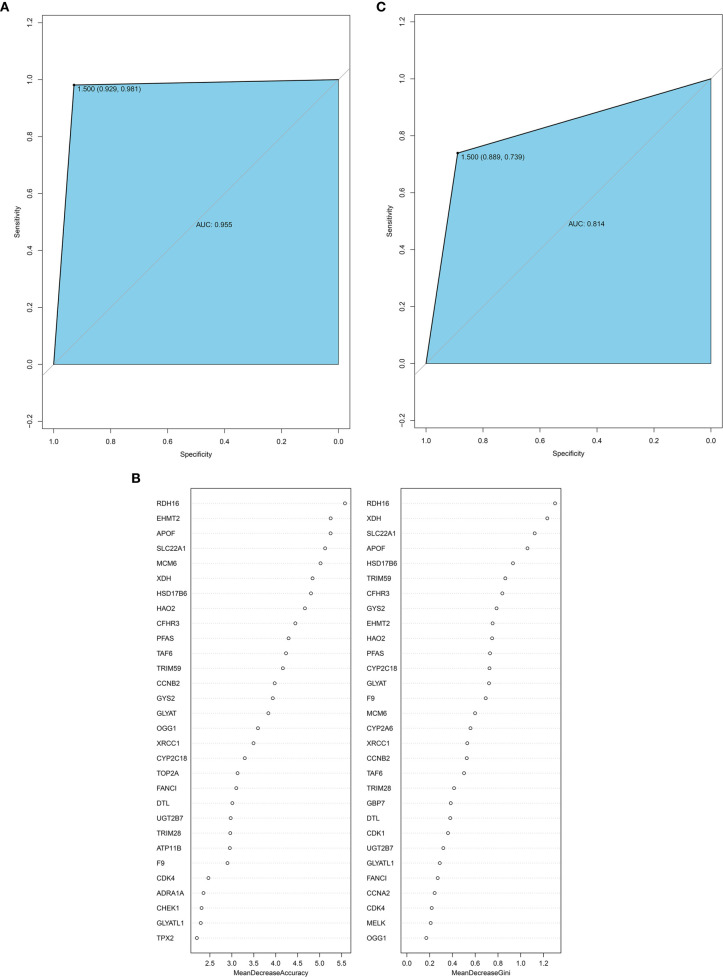
**(A)** ROC curve for the GSE131329 dataset; **(B)** 30 hub genes from the random forest classifier extracted through MDA and MDG; **(C)** ROC curve for the GSE81928 dataset. AUC, area under the curve; ROC, receiver operating characteristic; MDA, mean decrease accuracy; MDG, mean decrease Gini.

The random forest method can calculate the importance of a single feature and screen the feature against the selected dataset. Therefore, we used the 114 hub genes as the feature, HB and normal as the variables, and the GSE131329 dataset as the training set to construct the model. The receiving operator curve (ROC) of the GES131329 training set is shown in **Figure 8**. The area under the curve was 0.955. The mean decrease accuracy (MDA) of the random forest model was positively correlated with the predictive variable, and the mean decrease Gini (MDG) is positively correlated with the most important variable (20). Therefore, 30 hub genes were established using MDA and MDG (**Figure 8**). Furthermore, a total of 35 core genes of HB were obtained by cross-comparison with InnateDB database (**Supplementary Table 9**). The random forest model was then validated using the independent GSE81928 dataset, which was also contained the 114 hub genes. The area under the ROC curve was 0.814 (**Figure 8**).

### Validation of HB Biological Markers Through InnateDB Cross-Comparison

We selected the immune-related hub gene, containing 35 genes, as the HB innate immune core genes and compared it with the immune-related genes present in the InnateDB database. We obtained 10 core genes: *CDK1*, *TOP2A*, *ADRA1A*, *FANCI*, *XRCC1*, *TPX2*, *CCNB2*, *CDK4*, *GLYATL1*, and *CFHR3*. For nine genes at least one molecular interaction was annotated in the InnateDB database, except *GLYATL1*. These interactions were mainly protein-protein and DNA-protein, as shown in **Supplementary Figures 1**–**9**.

## Discussion

In this study, we established for the first time a diagnosis classifier model based on the random tree method for HB using 114 hub genes. We also validated the classification efficiency of this model using an independent dataset. Consequently, this model may contribute to improving the diagnosis of HB. We also performed GO and KEGG analyses, revealing that the identified hub genes were mainly involved in the p53 pathway and cell cycle. We also identified 10 core genes by cross-referencing our analysis with the InnateDB database. Among the 10 core genes, the molecular interactions for 9 genes were annotated, which may provide new therapeutic targets.

Among the 10 identified core genes, *CDK1* and *CDK4* were previously reported to be associated with HB (21, 22). *CDK1* and *CDK4* both belong to the family of cyclin-dependent kinases (CDKs). CDK complexes are critical regulatory enzymes that drive the transition of different phases of the cell cycle and ensure successful cell division through their activity (23). Almost all malignant cells exhibit some features that derange the normal controls over the cell cycle (24). Therefore, various drugs targeting different CDKs have been developed and have been applied in the clinic over the past decades. *CDK1* can bind to different cyclins and regulate all the steps required for cell division (25). For this reason, *CDK1* is essential for mammalian cell proliferation (21) and is the only CDK that can initiate mitosis (26). *CDK1* is a key determinant of mitotic progression and thus it is also a pivotal tumorigenic event. It has been reported that treatment with a *CDK1* inhibitor could decrease tumor growth of HB and prolong the survival rate in an HB murine model (21). Therefore, *CDK1* is considered an ideal target for HB treatment. *CDK4* can mediate the transition from the G0 or G1 phase into the S phase of the cell cycle (27). The activity of *CDK4* is primarily controlled by its association with D-type cyclins, with cyclin D1 being the best characterized. Kim et al. revealed that *CDK4* and cyclin D1 were significantly overexpressed in HB tissues compared with normal tissues (22). They also suggested that *CDK4* may be correlated with tumorigenesis, tumor recurrence, and metastasis of HB. Although there is still no available *CDK4* inhibitor for HB, multiple selective *CDK4* inhibitors targeting other types of cancer have been used in the clinic. The progression-free survival rate of patients with estrogen receptor-positive breast cancer can improve when *CDK4/6* inhibitors are added to antiestrogen therapy (28). Therefore, the role of *CDK4* in HB progression and treatment requires further studies.

The role of the other 8 core genes in HB has never been reported before. Among them, 6 genes have been reported to be associated with hepatocellular carcinoma (HCC). TOP2A was one of the top 20 genes with the highest degree of interaction in the PPI network complex. *TOP2A* encodes a DNA topoisomerase that controls and alters the topologic states of intertwined DNA during anaphase. Therefore, *TOP2A* is involved in chromosome condensation and chromatid separation (29). Overexpression of *TOP2A* is correlated with a more aggressive tumor phenotype, microvascular invasion, and early age onset of HCC (30). Moreover, *TOP2A* has also been a valuable prognostic marker for tumor advancements, recurrences, and predictors of poor survival in a variety of cancers, such as breast, ovarian, colon, and small cell lung cancer (29). *ADRA1A* encodes the alpha-1 adrenergic receptor subtype with catecholamines ligands (31), which is located on chromosome 8p (32). *ADRA1A* can stimulate the sympathetic nervous system to compete with some functions (33). It was reported by Chen et al. that the mean methylation level of the *ADRA1A* promoter region was significantly increased in HCC tissues compared with the normal tissues (32). They also demonstrated that the mean methylation levels of the *ADRA1A* gene in HCC samples were not only associated with clinical characteristics but could also discriminate between HCC tissues and adjacent normal tissues, thus being suitable as a diagnostic marker. *XRCC1* is a DNA repair gene that plays a crucial role in maintaining genomic integrity and stability and in the pathogenesis and carcinogenesis of various type of cancer (34). *XRCC1* is significantly correlated with the number of tumors, tumor size, and location, and is also an independent risk factor for the poor prognosis of HCC (34, 35). *TPX2*, a nuclear proliferation microtubule-associated protein, is essential for spindle formation and stabilizes spindle microtubules (36). The overexpression of *TPX2* induces abnormal centrosome amplification and aneuploidy formation, leading to malignant transformation of cells (37). Multiple studies have shown that the expression levels of *TPX2* were significantly upregulated in HCC tissues compared with the adjacent normal tissues (36–38). They also confirmed that *TPX2* may improve the viability of HCC cells and inhibit cell apoptosis. However, knockdown of *TPX2* expression or TPX2 inhibition could reduce the migration and invasion ability of HCC cells. *CCNB2* was one of the top 20 genes with the highest degree of interaction in the PPI network complex. *CCNB2* belongs to the B-type cyclin family and regulates the activity of CDKs by binding to them during the cell cycle (23). The overexpression of *CCNB2* was positively correlated with tumor number, tumor size, tumor thrombus, and metastasis of HCC, which may contribute to the poor prognosis of HCC patients (39–41). However, *CCNB2* knockdown could slow cell growth and promote apoptosis of HCC cells, indicating that *CCNB2* may be a novel treatment marker (41). *CFHR3*, a member of the human factor H protein family, is a negative complement activation regulator, which is an essential component of the innate immune system (42). The expression level of *CFHR3* in HCC tissues was lower than that in normal tissues (43). In addition, the expression level of the *CFHR3* gene was the highest in the liver than in other organs (44). *CFHR3* is correlated to the HCC stage. In addition, the overall survival of patients affected with HCC was significantly better when *CFHR3* was highly expressed than when its expression was low (43, 44). Therefore, *CFHR3* may be a novel prognostic biomarker for HCC. Although these 6 genes were never reported in the context of HB, our bioinformatics analysis suggests that they deserve further attention as potential targets in HB.

*FANCI* and *GLYATL1* have never been reported in either HB or HCC. However, their abnormal expression has been found in other tumor types. *FANCI* has a key role in the Fanconi anemia DNA repair pathway, where it forms a heterodimer with *FANCD2* and recruits DNA repair proteins to promote the interstrand cross-link DNA damage repair (45). Moreover, *FANCI* may promote cellular metabolism when it is not needed for DNA repair, according to a recent study (46). *FANCI* mRNA and protein were both found to be overexpressed in lung adenocarcinoma tumor tissues compared with adjacent normal tissues (47). It was demonstrated that the expression level of *FANCI* was positively associated with lymphatic metastasis and distant metastasis of lung adenocarcinoma tumor, whereas knockdown of *FANCI* decreased lung adenocarcinoma tumor cell proliferation and invasion *in vitro*. *FANCI* has also been reported to regulate breast cancer survival (48). These findings suggest that *FANCI* has a novel oncogenic role and may be useful as a prognostic biomarker and/or therapeutic target for different tumors. *GLYATL1* belongs to the glycine-N-acyltransferase gene family and is normally expressed in the liver and kidney (49). *GLYATL1* encodes an enzyme with phenylacetyl-CoA glutamine N-acyltransferase activity, which regulates mitochondrial ATP production, glycine availability, CoASH availability, and the detoxification of various organic acids (50). In a previous study, the expression of *GLYATL1* was higher in localized prostate cancers than in benign prostatic tissue and metastatic prostate cancer (49, 51). This study also demonstrated that *GLYATL1* may be associated with the grade of prostate cancer since the expression of *GLYATL1* was significantly high in low-grade tumors. Therefore, *GLYATL1* could be a potential early-stage biomarker. In addition, *GLYATL* was also found to be overexpressed in ER-negative compared to ER-positive breast cancer (52).

We also conducted GO enrichment and KEGG pathway analysis to identify pathways correlated with the hub genes. KEGG pathway analysis revealed that the largest number of genes were enriched in the cell cycle, including 13 hub genes. Most of them, including *CDK1* (21), *CDK4* (27), *BUB1* (53), *BUB1B* (54), *CCNA2* (55), *CCNB1* (56), *CCNB2* (39), *CDC6* (57), *MAD2L1* (58), *MCM6* (59), and *PLK1* (60) have been already reported to be associated with cell cycle-related proliferation and tumor differentiation. GO analysis further showed that hub genes are involved in different cell cycle-related processes, including mitotic nuclear division, cell division, chromosome separation, sister chromatid cohesion, microtubule cytoskeleton organization involved in mitosis, and DNA integrity checkpoint. Furthermore, GO enrichment and KEGG pathway analysis also demonstrated that the hub genes were associated with the p53 signaling pathway, a tumor suppression pathway through a variety of responses, including cell-cycle arrest, apoptosis, senescence, and DNA repair (61, 62), suggesting that the p53 signaling pathway is also involved in the cell cycle. It was reported that p53 gene mutations may contribute to the development of sporadic HB (63). Moreover, hepatic p53 expression could cause lysis of implanted hepatoblastoma cells in a chimeric mouse (64). Although p53 may play a crucial role in HB development, the specific mechanism needs further studies. Taken together, based on the GO and KEGG analyses, we suggest that targeting the cell cycle could be a potential strategy for HB therapy. Compared with the traditional clinical manifestations, imaging, AFP and other diagnostic methods, our study considered the underlying genetic dysregulations. Genes are more objective and stable; thus, they may not be beneficial for early diagnosis.

## Conclusion

In the present study, we established a 114 genes random forest classifier for HB and identified 10 core genes. These 10 core genes are closely related to the progression and prognosis of cancers and thus are also potential therapeutic targets. Our classifier model and the identified core genes may give novel insight into the diagnosis and development of therapeutic options for HB.

## Data Availability Statement

The datasets presented in this study can be found in online repositories. The names of the repository/repositories and accession number(s) can be found in the article/**Supplementary Material**.

## Author Contributions 

RS performed the data analyses and wrote the manuscript. SL, KZ, and MD contributed significantly to data analyses and manuscript revision. LL conceived and designed the study. All authors contributed to the article and approved the submitted version.

## Funding

This study was supported by grants from the Special and Key Projects in the Pediatrics of Beijing Hospitals Authority and Pediatric Collaborative Development (No. XTZD20180302) and Fundamental Research Funds for the Central Universities (No. 3332019166).

## Conflict of Interest

The authors declare that the research was conducted in the absence of any commercial or financial relationships that could be construed as a potential conflict of interest.
