# Supplementary figures and images for: Identification of Ten Core Hub Genes as Potential Biomarkers and Treatment Target for Hepatoblastoma
Source: Front Oncol. 2021 Apr 1;11:591507. doi: 10.3389/fonc.2021.591507 (PMC8047669; doi:10.3389/fonc.2021.591507)

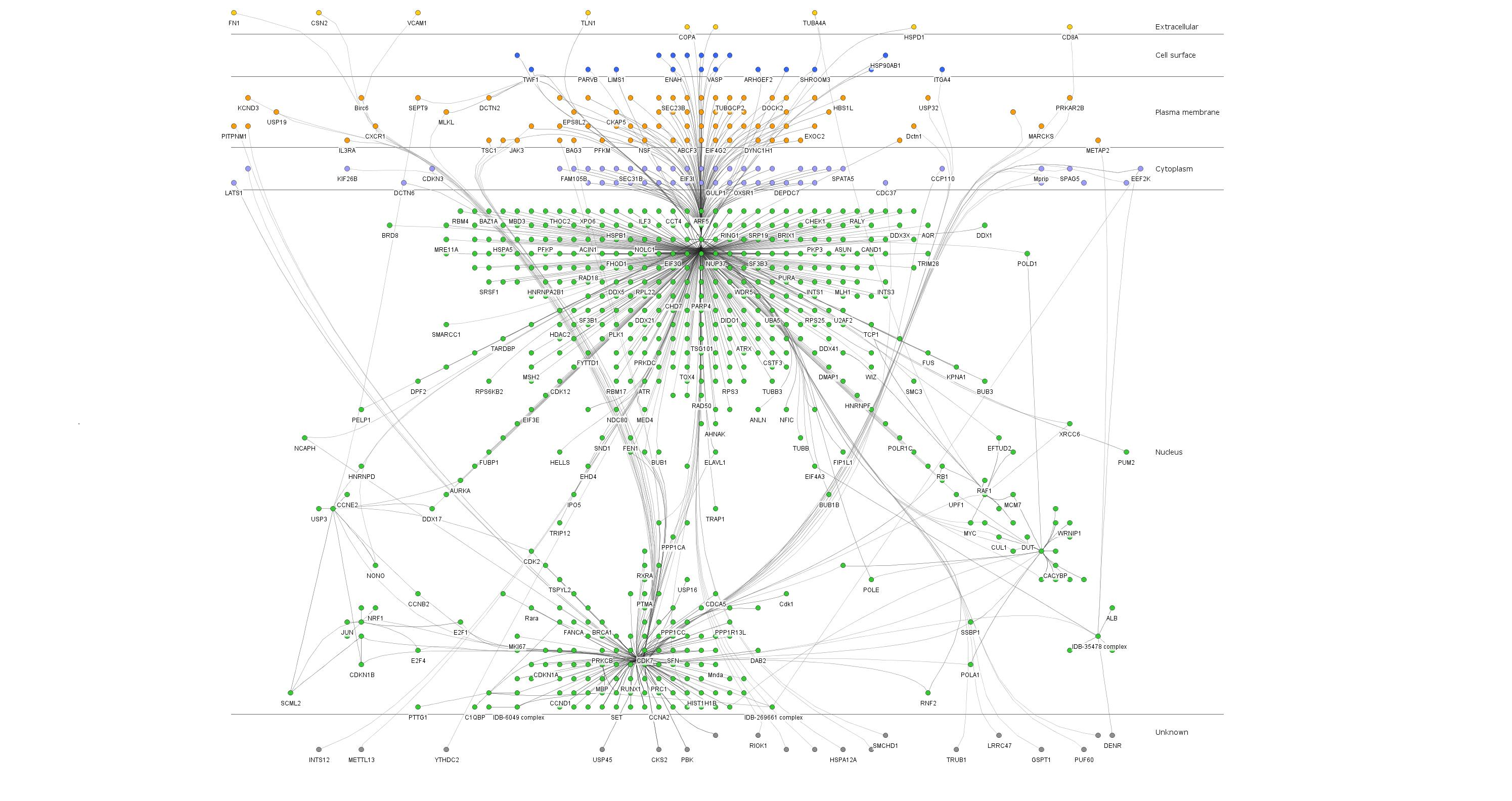

Supplement: Supplementary file 1 [file Image_1.tif]

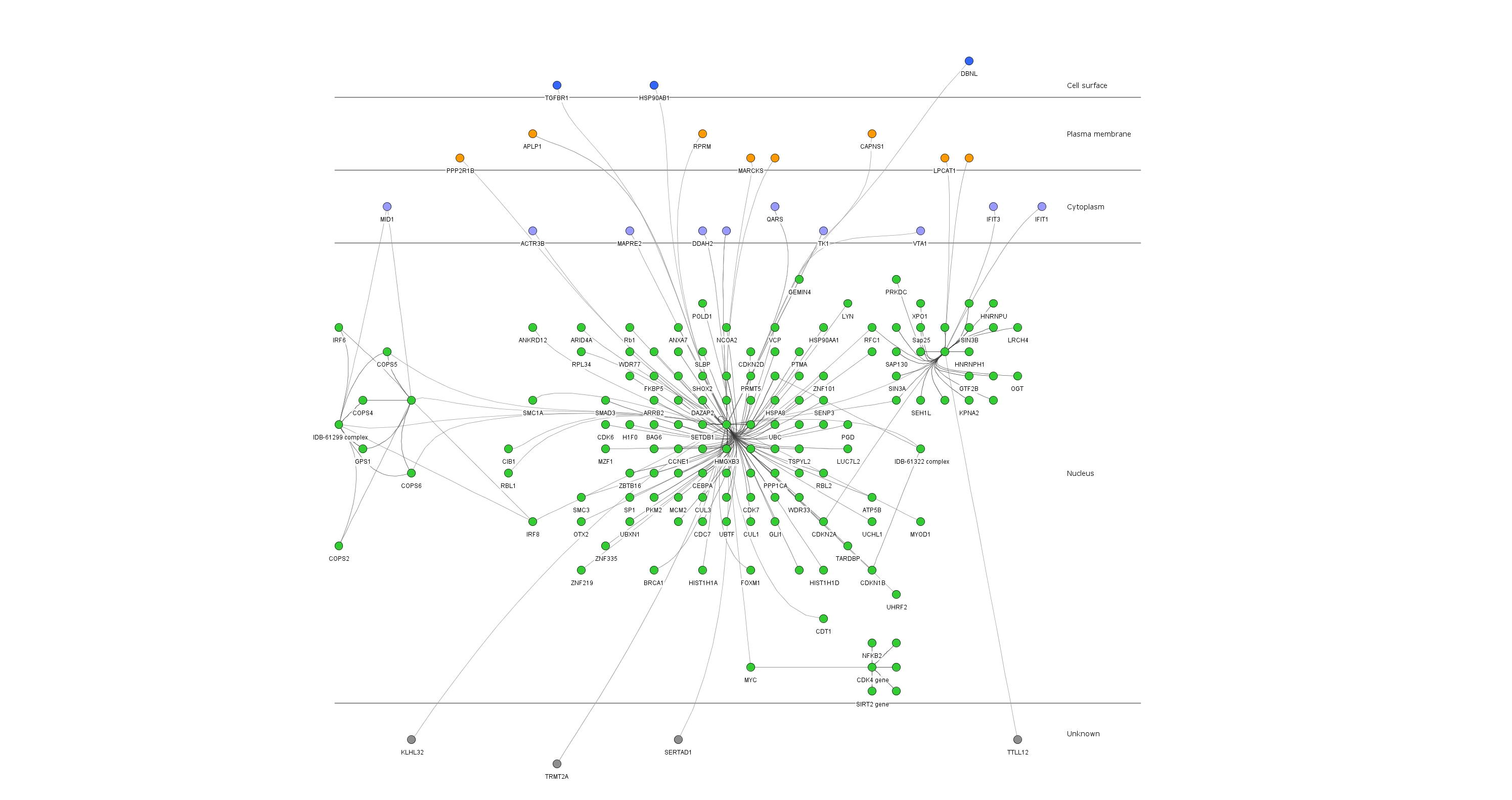

Supplement: Supplementary file 2 [file Image_2.tif]

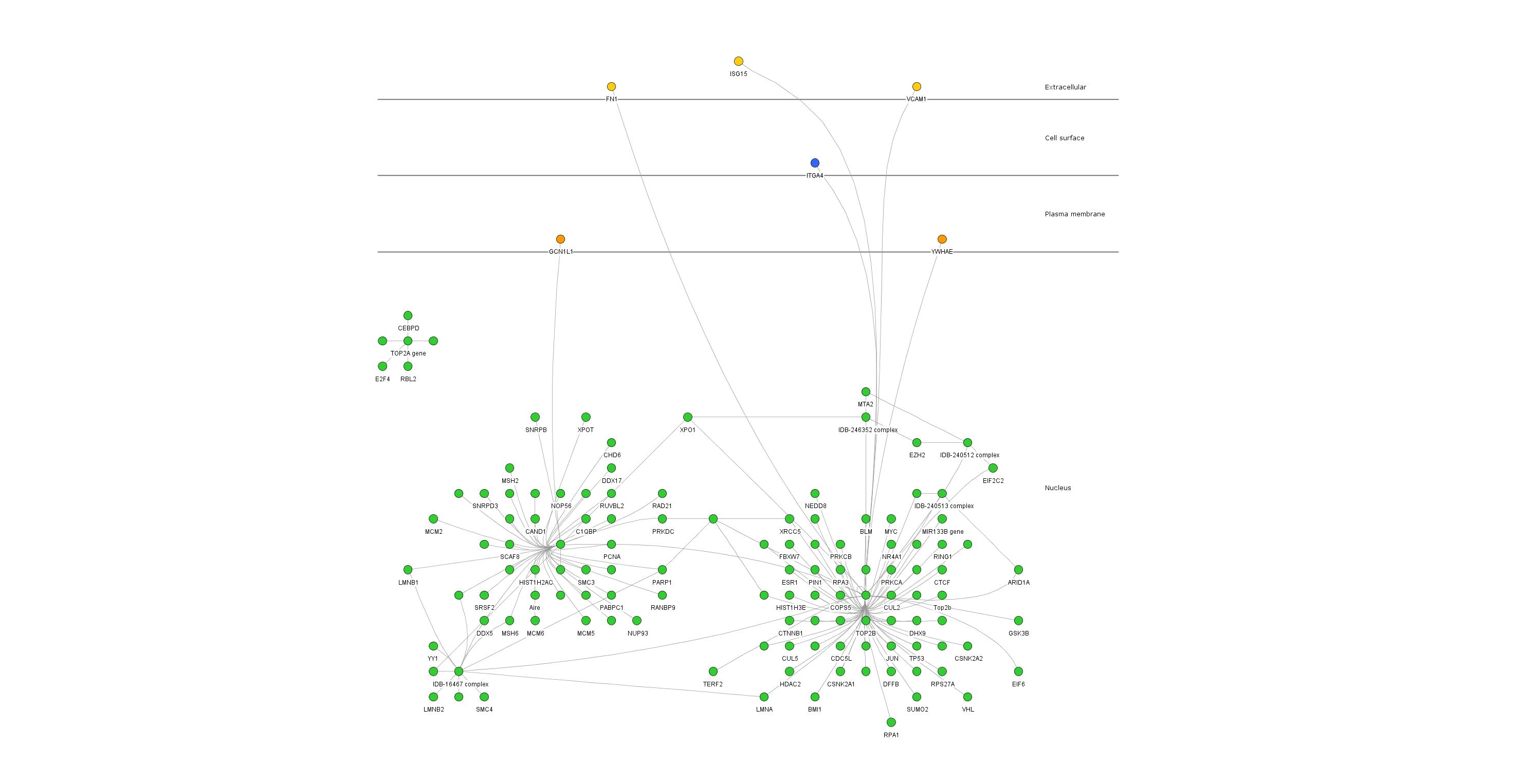

Supplement: Supplementary file 3 [file Image_3.tif]

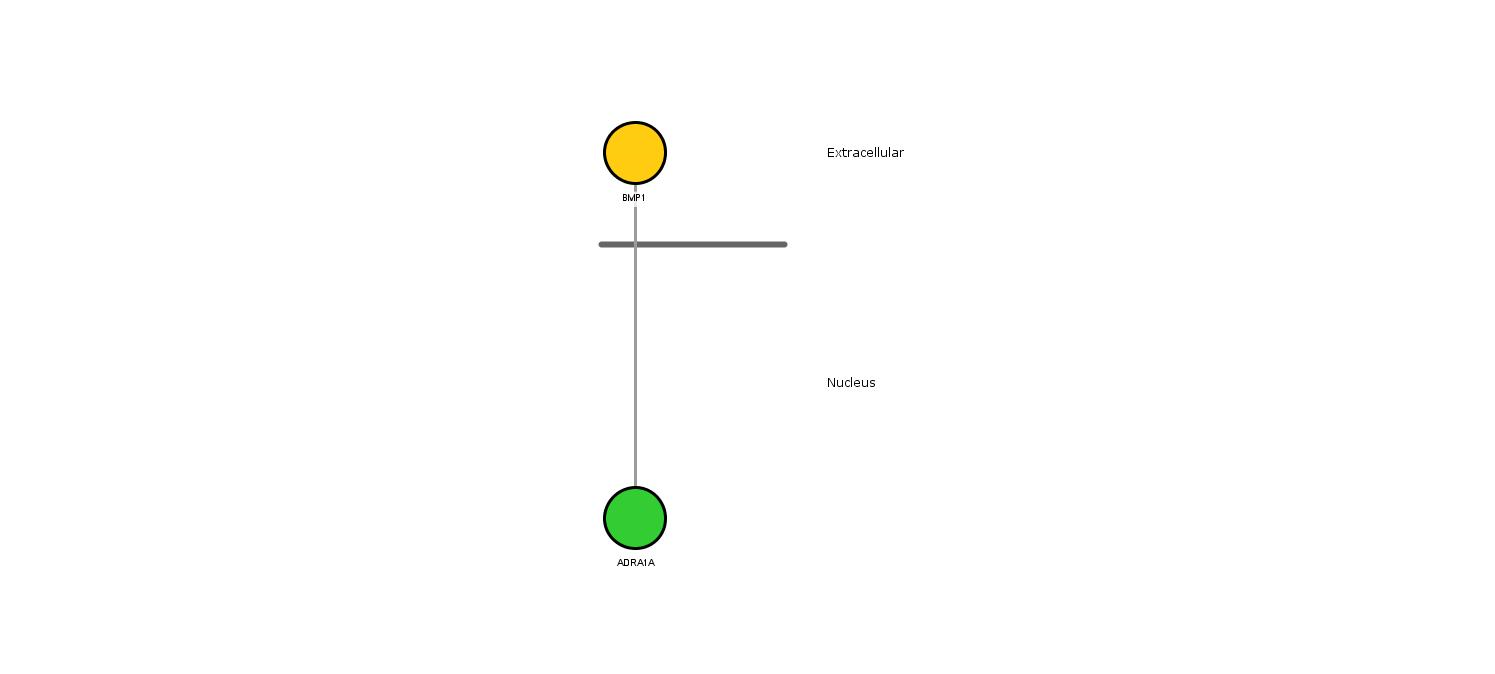

Supplement: Supplementary file 4 [file Image_4.tif]

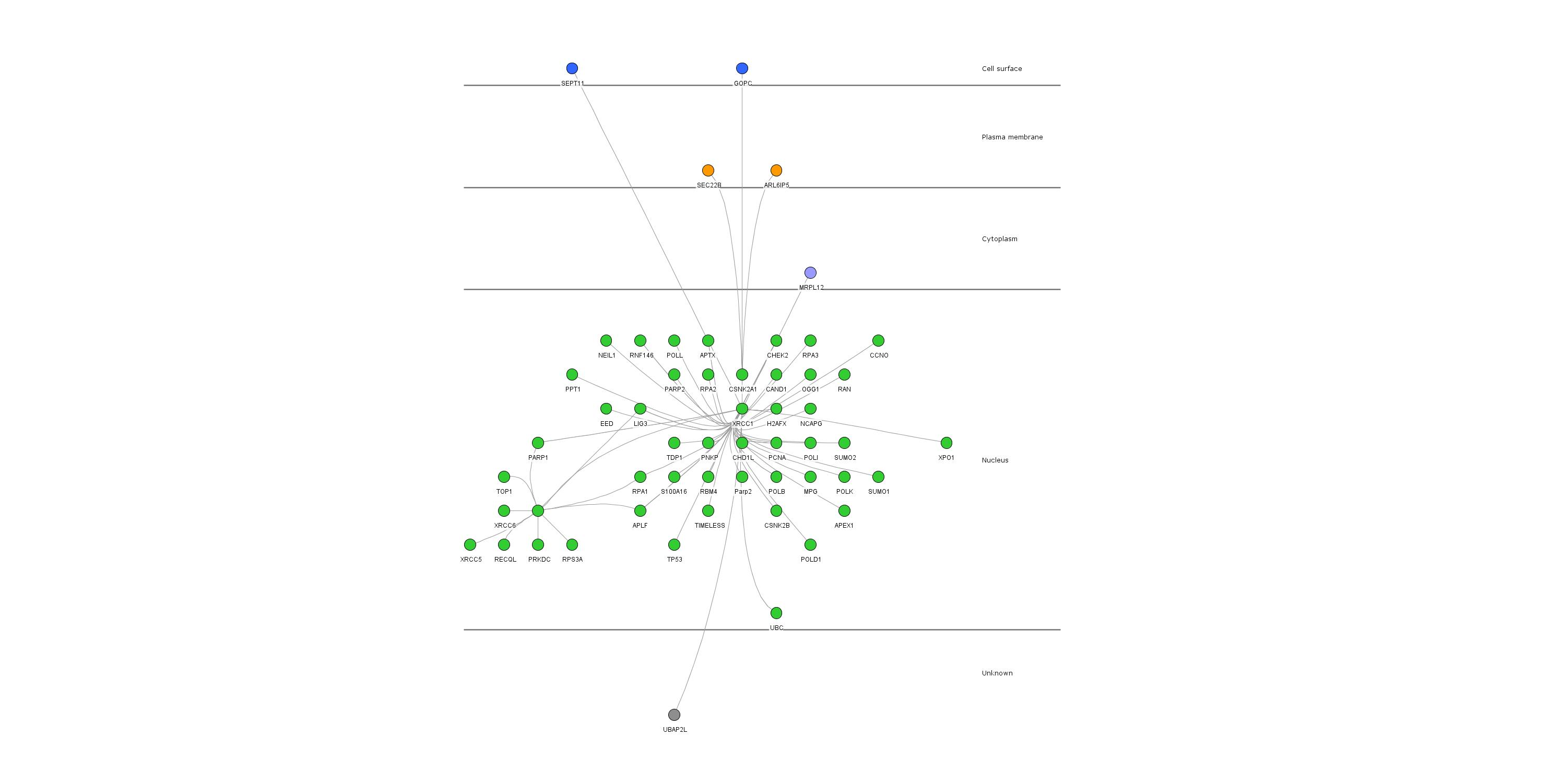

Supplement: Supplementary file 5 [file Image_5.tif]

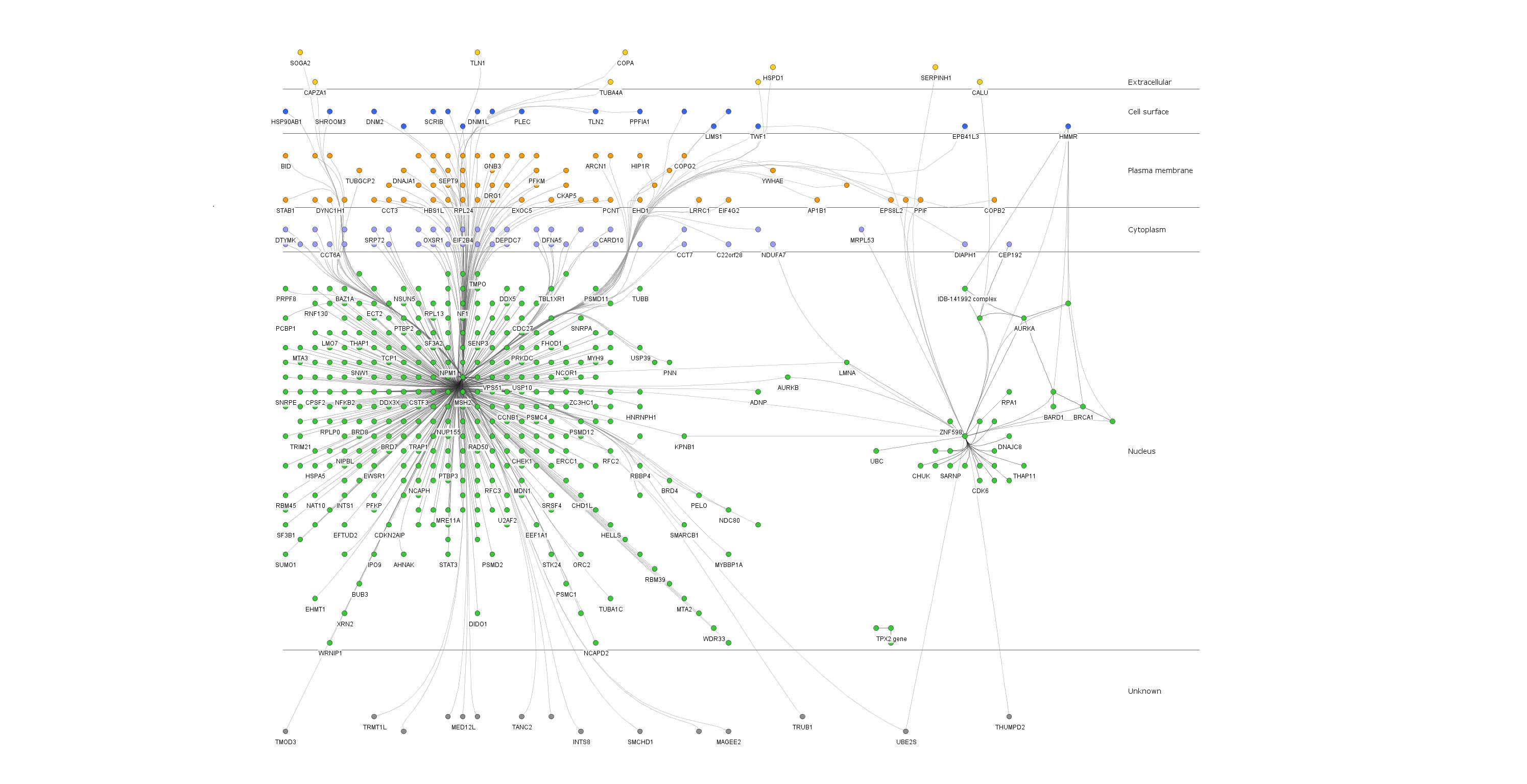

Supplement: Supplementary file 6 [file Image_6.tif]

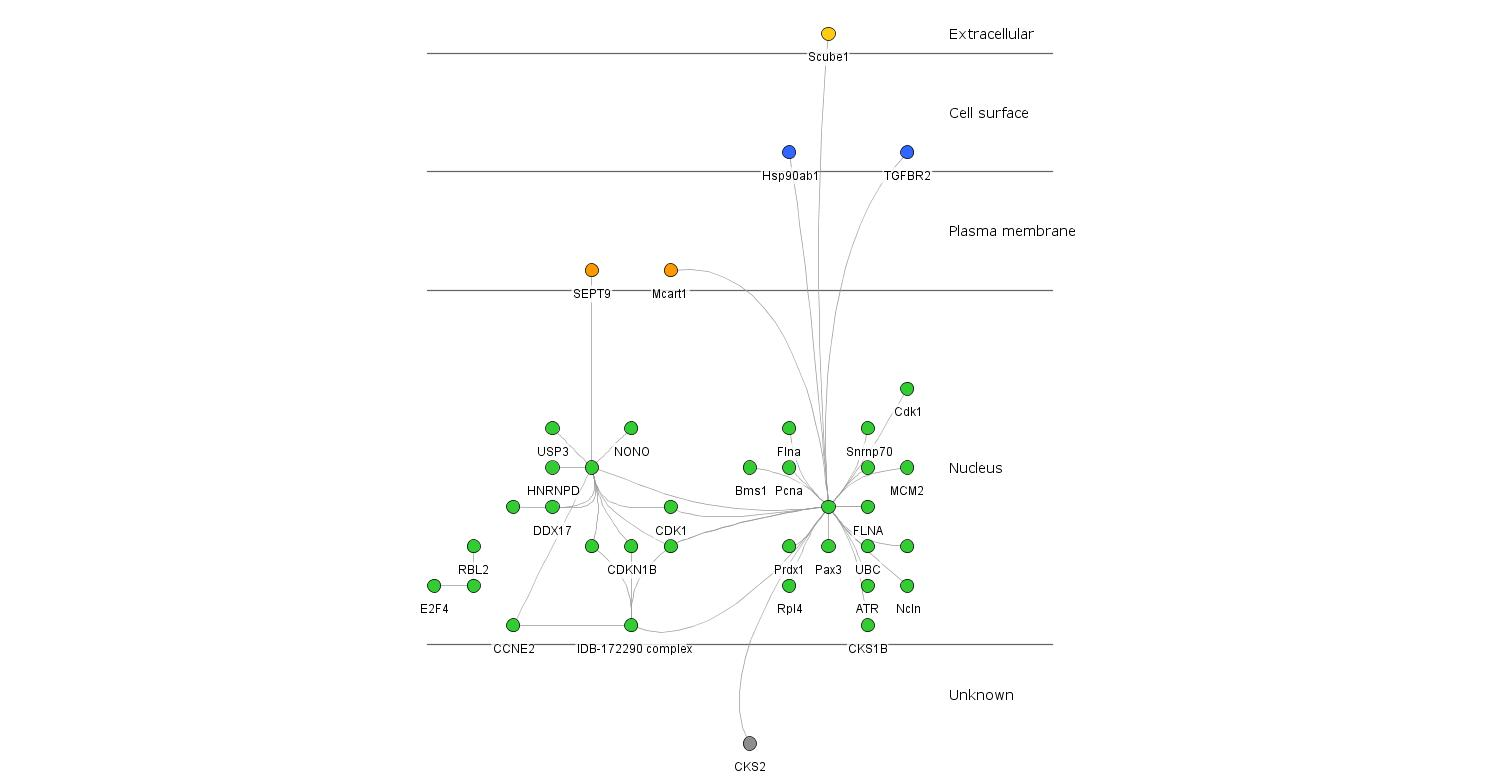

Supplement: Supplementary file 7 [file Image_7.tif]

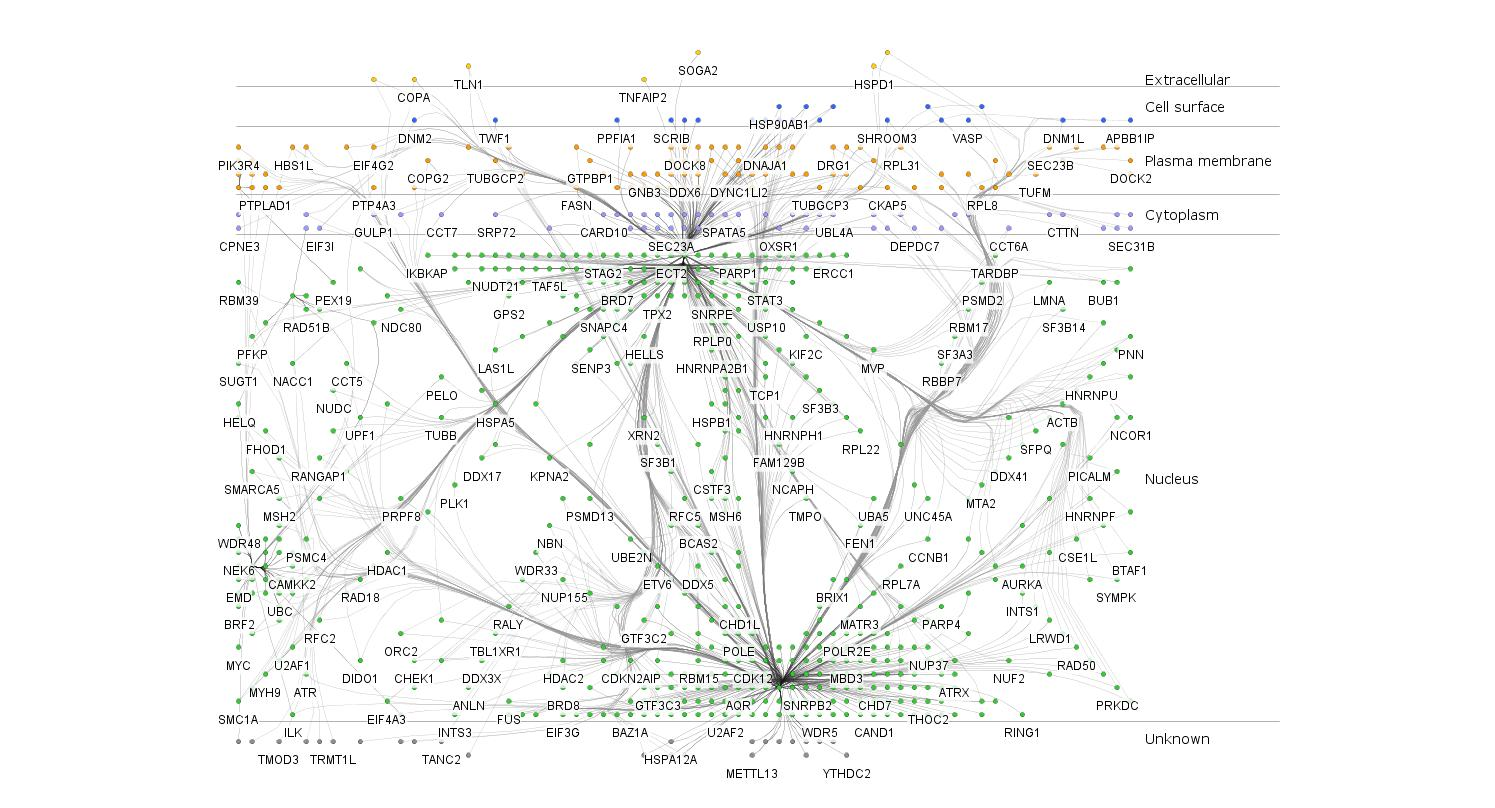

Supplement: Supplementary file 8 [file Image_8.tif]

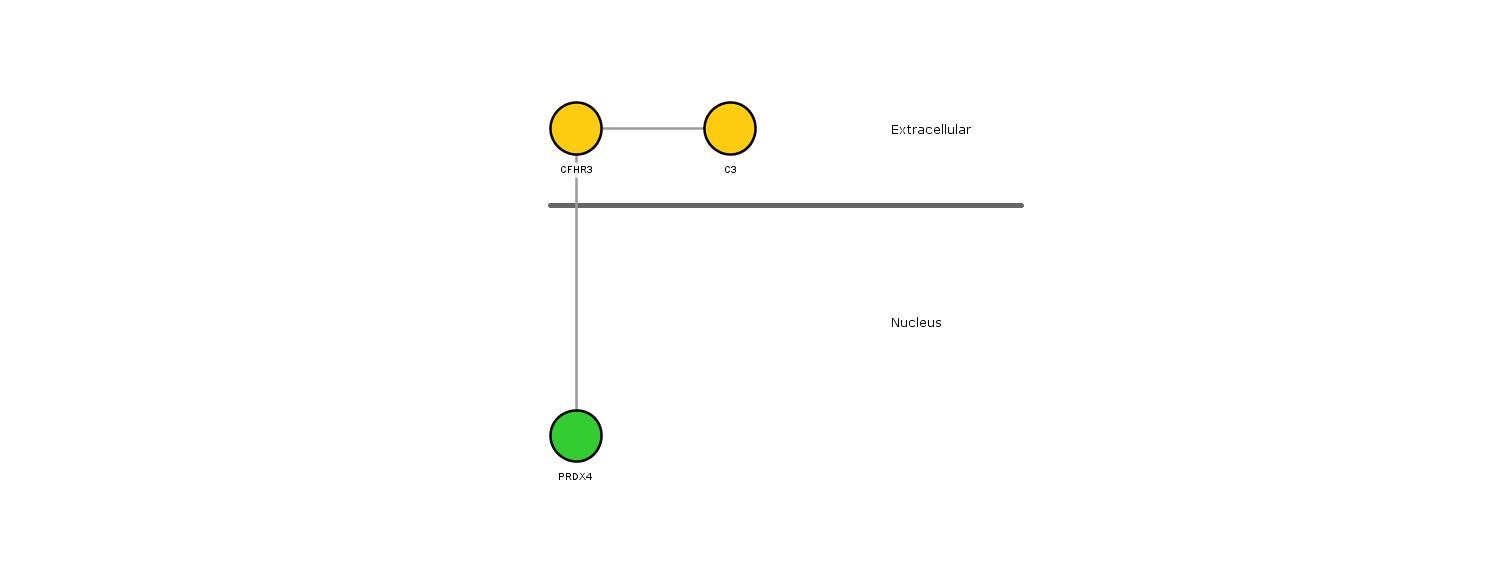

Supplement: Supplementary file 9 [file Image_9.tif]
